# Association between *FTO* rs9939609 genotype and breast cancer risk after bariatric surgery in the Swedish Obese Subjects study

**DOI:** 10.1038/s41598-026-51884-2

**Published:** 2026-05-06

**Authors:** Elin Langegård, Felipe M. Kristensson, Johanna C. Andersson-Assarsson, Markku Peltonen, Per-Arne Svensson, Peter Jacobson, Sofie Ahlin, Kajsa Sjöholm, Lena M. S. Carlsson, Magdalena Taube

**Affiliations:** 1 Department of Molecular and Clinical Medicine, Institute of Medicine, The Sahlgrenska Academy at University of Gothenburg, SOS Sekretariatet, Vita Stråket 15, 413 45 Gothenburg, Sweden; 2 Department of Microbiology and Immunology, Institute of Biomedicine, Sahlgrenska Academy, University of Gothenburg, Gothenburg, Sweden; 3 Department of Surgery, Region Västra Götaland, Sahlgrenska University Hospital/Östra, Gothenburg, Sweden; 4 Finnish Institute for Health and Welfare, Helsinki, Finland; 5 Institute of Health and Care Sciences, The Sahlgrenska Academy at University of Gothenburg, Gothenburg, Sweden; 6 NU Hospital Group, Department of Clinical Physiology, Region Västra Götaland, Trollhättan, Sweden

**Keywords:** Fat mass and obesity associated protein (*FTO*), Breast cancer, Obesity, Insulin, Cancer, Diseases, Endocrinology, Genetics, Oncology, Risk factors

## Abstract

The *FTO* rs9939609 SNP has been linked to higher BMI, insulin resistance, and breast cancer risk. Weight loss by bariatric surgery lowers breast cancer risk, particularly in women with baseline hyperinsulinemia, but it is unclear whether this effect varies by *FTO* genotype. In the Swedish Obese Subjects (SOS) study, 2596 women with severe obesity were followed for a median of 23.9 years to examine breast cancer incidence after bariatric surgery compared with usual care. Genotyping for rs9939609 was performed, and breast cancer events were identified via the Swedish National Cancer Registry. Among 135 breast cancer cases (77 in the control group and 58 in the surgery group), surgery was associated with a lower breast cancer incidence in carriers of the risk allele (TA/AA) (adjusted HR = 0.53 [0.34–0.83], *P* = 0.005), but not in non-carriers (TT) (adjusted HR = 1.19 [0.65–2.15], *P* = 0.573; *P* for treatment-genotype interaction = 0.031). These findings suggest that the *FTO* rs9939609 genotype may modify the association between bariatric surgery and breast cancer incidence. Trial registration: ClinicalTrials.gov, identifier: NCT01479452.

**Supplementary Information:**

The online version contains supplementary material available at 10.1038/s41598-026-51884-2.

## Introduction

Breast cancer represents one of the most frequently diagnosed malignancies and remains a leading contributor to cancer-related mortality among women worldwide^1^. Moreover, the global incidence of breast cancer is projected to rise, largely attributable to increasing rates of obesity and demographic shifts toward an older population^2,3^. Obesity not only constitutes a significant risk factor for breast cancer but also worsens the prognosis for those affected^4^. In contrast, weight loss in people with obesity has been associated with a decreased risk of cancer^5–8^. Bariatric surgery constitutes a clinically validated intervention for achieving substantial and sustained weight reduction^9,10^. Several studies have shown that bariatric surgery decreases the incidence of obesity-associated and overall cancer^5,8,11^. Our previous research has demonstrated a decreased risk of female-specific malignancies^12^, including breast cancer, following bariatric surgery^13^. While we demonstrated that the protective effect of bariatric surgery on cancer risk is most pronounced in women with hyperinsulinemia^12,13^, much remains to be understood about the mechanisms linking obesity and cancer risk^14^.

A substantial body of evidence indicates that genetic factors contribute to interindividual variability in weight-related traits, including body mass index and adiposity. Classic twin studies have demonstrated marked intrapair similarity in weight change under controlled negative energy balance conditions, supporting a heritable component of weight loss response^15,16^. Genetic variation may therefore influence not only susceptibility to obesity but also physiological adaptation to sustained weight reduction and the metabolic consequences of bariatric surgery.

The fat mass and obesity associated gene (*FTO*), identified as an obesity-related gene in 2007, is involved in several cellular processes including gene transcription, cell apoptosis, and metabolism. Single nucleotide polymorphisms (SNPs) in intron 1 of the *FTO* gene have been associated with obesity in individuals of European descent^17^, and a SNP in the *FTO* locus has been associated with differences in maximal weight loss following bariatric surgery^18^. Among these variants, rs9939609 is the most extensively studied and the A allele is common in populations of European ancestry, with a reported allele frequency of approximately 40%, underscoring its potential population-level relevance^17^. Carriers of the A-allele have been reported to exhibit higher BMI and increased food intake^17,19^, as well as an elevated risk of developing type 2 diabetes (T2D)^20^ and insulin resistance^21^. In addition to its effects on metabolism, rs9939609 has been associated with increased breast cancer risk^22,23^, suggesting a potential link between genetic predisposition, metabolic regulation, and cancer risk in the context of weight reduction.

Previous studies on the association between the rs9939609 genotype and breast cancer risk have primarily relied on case–control studies^24^. To our knowledge, no prospective studies have investigated the long-term effects of bariatric surgery on breast cancer risk and the influence of rs9939609 on treatment benefit in women with obesity. The aim of this study was to examine the association between rs9939609 and incident breast cancer in women treated with bariatric surgery or conventional obesity care in the Swedish Obese Subjects (SOS) study during up to 33 years of follow-up.

## Methods

### Study design and participants

The SOS study has been described in detail elsewhere^10,25,26^. In brief, the SOS study is a non-randomized prospective controlled intervention study originally designed to examine the long-term associations between intentional weight loss and mortality and morbidity. The study includes 4047 individuals, 2010 who underwent bariatric surgery and 2037 matched controls (matched on 18 variables). The matched controls received routine nonsurgical obesity care at their primary healthcare centers. This care was not standardized and ranged from structured lifestyle interventions to little or no active treatment. The surgery group was formed from individuals electing surgery and the surgical techniques used were vertical banded gastroplasty, non-adjustable or adjustable gastric banding, and gastric bypass^26^. The surgery and control groups had identical inclusion and exclusion criteria. Inclusion criteria: age 37–60 years and a BMI ≥ 38 kg/m^2^ for women and BMI ≥ 34 kg/m^2^ for men. The exclusion criteria were selected to exclude participants not suitable for surgery^25^. Seven regional ethics review boards approved the study protocol (Gothenburg, Linköping, Skåne, Stockholm, Umeå, Uppsala and Örebro). Written or oral informed consent was obtained from all participants. The study adhered to the principles outlined in the Declaration of Helsinki.

### Outcomes and follow-up

**Fig. 1:**
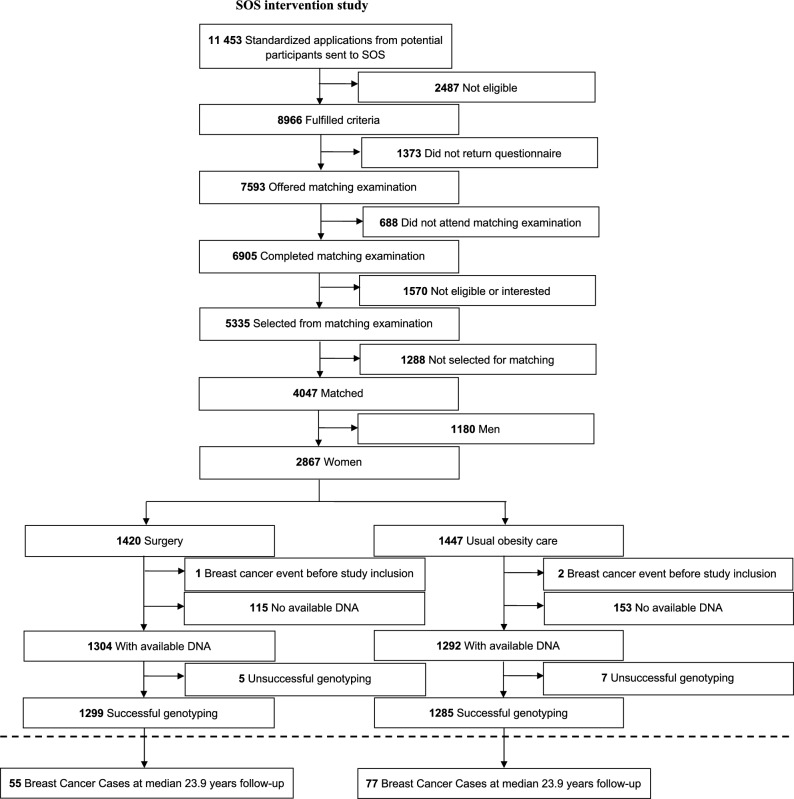
Study flowchart of participants in the Swedish Obese Subjects (SOS) study and breast cancer events during follow-up.

The current report includes women in the SOS study; 1420 in the surgery group of which 970 underwent vertical banded gastroplasty, 260 gastric banding, and 190 gastric bypass. The control group consists of 1447 women receiving standard obesity care. A flow diagram of participant recruitment and inclusion in this report is shown in Fig. 1.

Baseline characteristics were obtained through clinical examinations, questionnaires, and centralized blood biochemistry analyses. Baseline alcohol intake was assessed using a validated, semi-quantitative dietary questionnaire capturing habitual intake over the previous 3 months^27^, while smoking was identified by a positive response to the query “Do you smoke daily?”. Additional clinical examinations were conducted at 0.5, 1, 2, 3, 4, 6, 8, 10, 15, and 20 years, and blood samples for biochemical analysis were collected at 2, 10, 15, and 20 years. Information regarding breast cancer diagnosis was obtained from the Swedish National Cancer Registry using ICD7 code 170. The Swedish National Cancer Registry has over 95% coverage for all malignant tumors of which 99% are morphologically verified^28^. Data on death and emigration were obtained by cross-checking social security numbers from the SOS database with the Cause of Death Registry and the Registry of the Total Population.

### Genotyping

The *FTO* rs9939609 SNP was genotyped using a TaqMan allelic discrimination PCR assay (Applied Biosystems, Thermo Fisher Scientific, Waltham, Massachusetts, USA). The genotyping was performed using the Viia7 Real-Time PCR system (Applied Biosystems) with the following thermal cycling conditions: 30 s at 60 °C, 5 min at 95 °C followed by 40 cycles at 95 °C for 15 s and 60 °C for 60 s followed by a last step of 60° for 30 s. Data analysis was performed in the Viia7 Real-Time PCR system software v1.3.

### Statistics

Data were censored if the woman emigrated from Sweden, changed treatment group, died before the end of follow-up due to reasons other than breast cancer, or remained event-free by the end of follow-up (December 31st, 2020). Thus, time at risk in the time-to-event analyses was calculated accounting for loss to follow-up. Three participants were excluded due to a breast cancer diagnosis before inclusion in the study, two in the control group and one in the surgery group. A per protocol approach was used in all analyses, including all participants in their original study group until bariatric surgery was performed in the control group or an operation which reinstated normal anatomy was performed in the surgery group, at which point they were censored from the analysis.

Baseline characteristics are reported as mean values and standard deviations or count and percent. Hardy–Weinberg equilibrium was calculated on genotype frequencies. Genotypes were analyzed using a dominant model comparing A-allele carriers (TA/AA) with non-carriers (TT).

Differences in changes of BMI and insulin over time between the surgery and control groups stratified by genotype were analyzed with multilevel mixed-effect regression models. Observations were considered nested within the individuals, and the statistical tests and confidence intervals were calculated controlling for the repeated measurements.

Kaplan–Meier estimates of the cumulative incidence were calculated. The Cox proportional hazard regression model was used to estimate the effect of genotype (TT vs. TA/AA) on breast cancer events in the control group only. Furthermore, the Cox proportional hazard regression model was used to estimate the effect of bariatric surgery on breast cancer events compared to the control group and stratified by genotype (TT vs. TA/AA) as well as stratified by insulin levels. Results are presented as hazard ratios (HR) with 95% confidence intervals (CI). Analyses were adjusted for age, BMI, smoking and alcohol intake at baseline.

The proportional-hazards assumption was evaluated by assessing the interaction between treatment and the logarithm of time.

Sensitivity analyses included additional adjustment for menopausal status and hormone replacement therapy (HRT) use at baseline and exclusion of individuals with medically treated diabetes at baseline.

Interaction effects were evaluated by including the treatment and rs9939609 genotype product-term in the Cox proportional hazards regression model, fitting the data to a dominant model. The rs9939609 genotype was additionally evaluated using an additive genetic model (coded 0, 1, 2 according to number of A alleles) as a sensitivity analysis. Corresponding hazard ratios were estimated using Cox proportional hazards models analogous to the primary analysis.

All statistical tests were two-tailed with a *P* value threshold less than 0.05 considered statistically significant. Statistical analysis was carried out using R version 4.2.1 (R Foundation for Statistical Computing, URL: https://www.R-project.org/) and STATA version 15.1 (StataCorp, 2017. Stata Statistical Software: Release 15. College Station, TX: StataCorp LLC).

## Results

DNA samples were available from 2596 women, of whom 2584 (99.5%) were successfully genotyped. Among these, 1881 individuals were carriers of the A risk allele (TA/AA), while 703 were non-carriers (TT). The allele frequencies for rs9939609 were 0.517 for the T allele and 0.483 for the A allele, with genotype distribution consistent with Hardy–Weinberg equilibrium (*P* value = 0.33).

### Baseline characteristics and BMI during follow-up

**Fig. 2:**
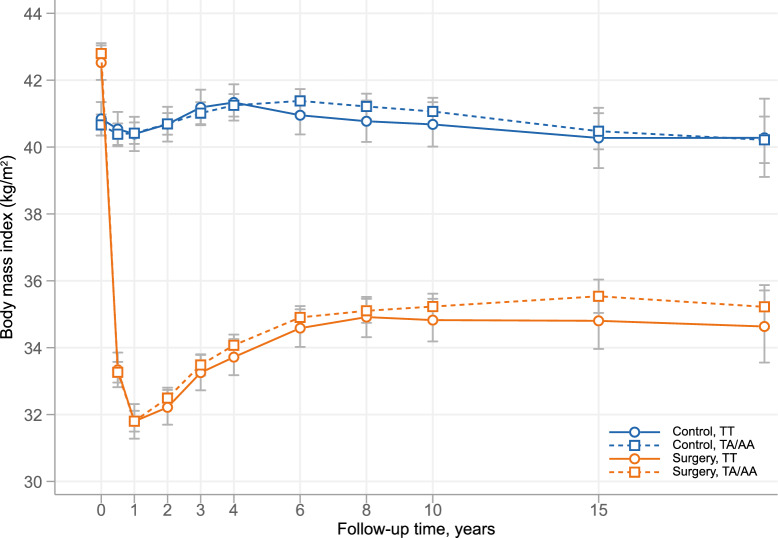
Changes in body mass index in women from the SOS study, according to treatment group and *FTO* rs9939609 genotype. Error bars represent 95% CI.

Baseline characteristics of A-allele carriers and non-carriers, stratified by treatment, are shown in Table 1. Among both A-allele carriers and non-carriers, women in the surgery group were on average 1 year younger than those in the control group. However, the bariatric surgery group had a higher weight, BMI, and a higher prevalence of smoking and diabetes, all known cancer risk factors. BMI changes were similar in A-allele carriers and non-carriers in both the bariatric surgery and control groups as shown in Fig. 2.

**Table 1:** Baseline characteristics of women in the Swedish Obese Subjects study.

	TA/AA genotype (n = 1881)		TT genotype (n = 703)	
	Control (n = 923)	Bariatric surgery (n = 958)	Control (n = 362)	Bariatric surgery (n = 341)
Age (y)	48.6 ± 6.3	47.0 ± 6.0	48.8 ± 6.2	47.4 ± 6.0
Body mass index (kg/m^2^)	40.7 ± 4.6	42.8 ± 4.3	40.8 ± 4.4	42.5 ± 4.1
Weight (kg)	110.4 ± 14.7	115.9 ± 13.7	110.8 ± 13.8	115.0 ± 13.2
Diabetes, n (%)	107 (11.6)	138 (14.5)	31 (8.6)	52 (15.3)
Fasting insulin (mU/L)	16.4 ± 10.0	20.0 ± 13.8	16.3 ± 8.9	19.2 ± 10.1
Blood glucose (mmol/L)	4.9 ± 1.8	5.1 ± 2.0	4.8 ± 1.4	5.0 ± 1.7
Menopause, n (%)	323 (35.0)	276 (28.8)	137 (37.8)	109 (32.0)
Smokers, n (%)	184 (20.0)	255 (26.7)	61 (16.9)	90 (26.4)
Alcohol intake (g/day)	3.3 ± 5.2	3.2 ± 4.6	3.6 ± 5.0	3.1 ± 4.4
Previous cancer, n (%)	13 (1.4)	13 (1.4)	4 (1.1)	6 (1.8)
Breast cancer events during FU	57	34	20	24

Values are mean ± SD unless otherwise indicated. FU, follow-up.

### Breast cancer incidence and rs9939609 genotype in the SOS control group

Over a median follow-up period of 22.7 years (IQR = 16.6–26.8), a total of 77 breast cancer events were observed in the control group: 57 among A allele carriers (TA/AA) and 20 among non-carriers (TT). There was no significant difference in breast cancer incidence between the A-allele carrier group (TA/AA) and non-carriers (HR = 1.11 [0.67–1.85], *P* = 0.687) (Supplementary Fig. 1).

### Association between rs9939609 genotype and breast cancer risk after bariatric surgery

**Fig. 3:**
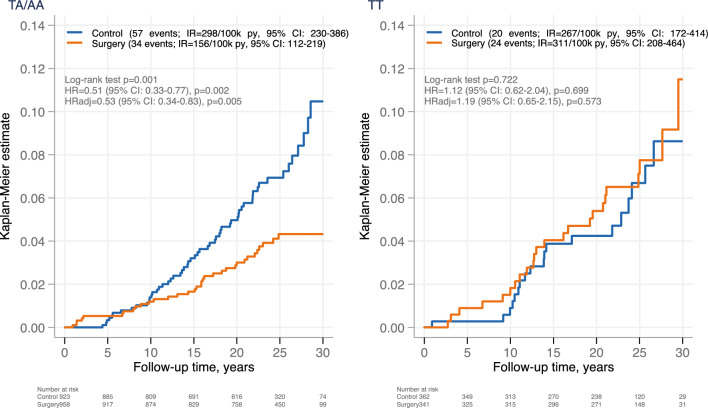
Cumulative incidence of breast cancer by *FTO* rs9939609 genotype (TA/AA vs. TT) in women from the SOS study, stratified by treatment.

Over a median follow-up of 23.9 years (IQR 20.1–27.1), 58 breast cancer events were observed in the surgery group: 34 in the risk allele (TA/AA) carrier group and 24 in the non-carrier (TT) group. To investigate if rs9939609 is associated with treatment effect, we compared the incidence of breast cancer between control and surgery patients, stratified by genotype (Fig. 3). In the risk allele (TA/AA) carrier group, surgery was associated with a lower incidence of breast cancer (HR = 0.51 [0.33–0.77], *P* = 0.002). In contrast, in the non-carrier (TT) group there was no significant difference in breast cancer incidence between the treatment groups (HR = 1.12 [0.62–2.04], *P* = 0.699, *P* for treatment-genotype interaction = 0.032). In the risk allele (TA/AA) carrier group, the control group had a lower incidence rate during the early follow-up as compared to the surgery group, whereas the surgery group had markedly lower rate later in the follow-up. The test of proportional hazards resulted in *P* = 0.065. Similar results were obtained after adjustment for age, BMI, smoking and alcohol intake (adjHR = 0.53 [0.34–0.83], *P* = 0.005, and adjHR = 1.19 [0.65–2.15], *P* = 0.573 for risk allele (TA/AA) carrier and non-carrier (TT) groups respectively, *P* for treatment-genotype interaction = 0.031). The associations remained essentially unchanged after additional adjustment for menopausal status and HRT use at baseline (Supplementary Fig. 2).

In sensitivity analyses using an additive genetic model, no clear linear dose–response relationship was observed between the number of A alleles and the reduction in breast cancer risk after bariatric surgery (Supplementary Fig. 3). Consistent with the primary analysis, the association between bariatric surgery and reduced breast cancer risk was observed among individuals carrying one or two A alleles; however, the per-allele interaction term did not reach statistical significance (adjusted *P* = 0.082).

### Association between rs9939609 genotype and breast cancer risk after bariatric surgery in groups stratified by baseline insulin levels

**Fig. 4:**
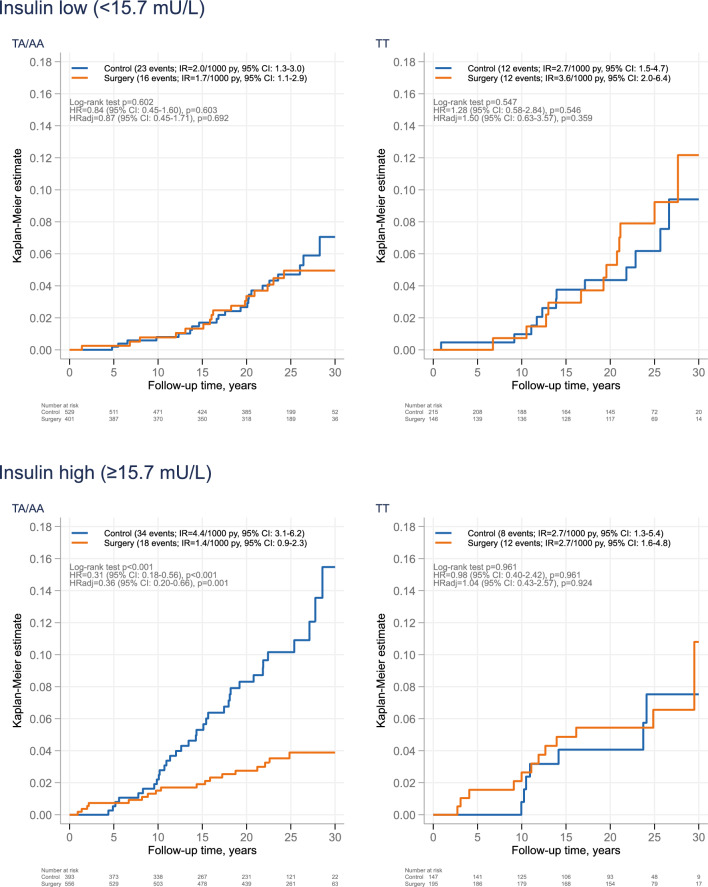
Cumulative incidence of breast cancer by treatment group in women from the SOS study, stratified by *FTO* rs9939609 genotype and baseline fasting insulin levels.

We subsequently stratified the treatment groups based on genotype (TA/AA vs. TT) and baseline insulin levels, using the median value (15.7 mU/L) as the cutoff (Fig. 4). Among women with baseline insulin levels above the median, bariatric surgery was associated with a reduced breast cancer incidence in carriers of the risk allele (TA/AA), with 34 events in the control group and 18 in the surgery group (HR = 0.31 [0.18–0.56], *P* < 0.001), while no association was found in non-carriers, with 8 and 12 events in the control and surgery groups, respectively (HR = 0.98 [0.40–2.42], *P* = 0.961; P for treatment-genotype interaction = 0.033). The results were similar after adjustment for age, BMI, smoking and alcohol intake with adjHR = 0.36 [0.20–0.66], *P* = 0.001, and adjHR = 1.04 [0.43–2.57], *P* = 0.924 for A-allele carriers and non-carriers, respectively; *P* for treatment-genotype interaction = 0.036. In contrast, among women with baseline insulin levels below the median, bariatric surgery was not associated with breast cancer incidence in either genotype group (23 vs. 16 events in TA/AA and 12 vs. 12 events in TT), in either unadjusted or adjusted analyses. There was no clear indication of violation of the proportional hazards assumption in any of the subgroups defined by insulin level and genotype (test of proportional hazard *P* > 0.10 for all comparisons). Although statistically significant associations were observed among TA/AA carriers with high baseline insulin levels, the number of events in some subgroups was modest, warranting cautious interpretation.

In a sensitivity analysis excluding individuals with medically treated diabetes at baseline, the results remained similar (Supplementary Fig. 4). Among women with baseline insulin levels above the median, bariatric surgery was associated with a reduced risk of breast cancer in TA/AA carriers (adjHR = 0.37 [0.20–0.69], *P* = 0.002), whereas no association was observed among TT carriers (adjHR = 0.97 [0.39–2.43], *P* = 0.949). However, the interaction test did not reach statistical significance (*P* = 0.062).

### Association between rs9939609 and insulin levels during follow-up

Throughout the follow-up period, insulin levels declined in both A-allele carriers and non-carriers within the surgical group, remaining below baseline values and reaching their lowest point at two years. In contrast, insulin levels in the control group remained relatively stable compared to baseline across both genotype groups (Supplementary Fig. 5). To assess the relationship between rs9939609 and changes in fasting insulin levels over time, mixed model analyses were performed. In analyses stratified by intervention group, no significant differences in fasting insulin levels during follow-up were observed between TT and TA/AA genotype carriers. In the surgery group, the adjusted mean difference in insulin (TT vs. TA/AA) was − 0.7 mU/L (95% CI − 1.7 to 0.2, *P* = 0.902), and in the control group 0.1 mU/L (95% CI − 0.9 to 1.1, *P* = 0.147).

## Discussion

In this non-randomized prospective controlled intervention study, we observed that bariatric surgery was associated with a 47% lower breast cancer incidence in women carrying the *FTO* rs9939609 risk allele (TA/AA), whereas no such association was observed in non-carriers (TT). Furthermore, in risk allele carriers with high baseline insulin levels, bariatric surgery was associated with an even greater reduction (64%) in breast cancer incidence.

These findings align with our earlier study, which indicated that the beneficial impact of bariatric surgery on breast cancer risk was most evident in women exhibiting elevated insulin levels at baseline^13^. Bariatric surgery is an effective method for achieving significant and sustained weight loss in individuals with obesity and has been associated with improvements in metabolic parameters, including reductions in circulating insulin levels^29^. As insulin has mitogenic, antiapoptotic and angiogenic effects^30,31^, we explored the association between rs9939609 and insulin levels during follow-up. Our analysis revealed no significant associations between genotype and insulin levels throughout the follow-up period. This suggests that the modifying effect of the rs9939609 genotype on treatment response is not mediated by changes in insulin levels. Alternatively, it could also indicate that insulin and rs9939609 influence breast cancer risk through separate pathways. Further studies are warranted to explore the relationship between the rs9939609 genotype and insulin more comprehensively.

In the overall cohort, the frequency of the A allele was slightly higher (0.517) than the general population’s frequency of 0.410, likely reflecting the established association with increased BMI^17^ and prevalence of obesity in the SOS cohort. No association between rs9939609 and breast cancer incidence was observed in the control group, a finding that should be interpreted in light of the high BMI within the SOS cohort and the established role of obesity as a risk factor for breast cancer. Thus, genotype-related differences may become more apparent in the setting of substantial weight reduction.

A previous meta-analysis reported no overall association between rs9939609 and breast cancer risk^32^. While this suggests that the variant does not confer a strong independent effect at the population level, the included studies did not account for adiposity-related stratification. More recent studies have suggested that associations between rs9939609 and breast cancer risk may be influenced by BMI, metabolic status, or tumour subtype^23,33,34^, indicating that the impact of this variant may be context-dependent rather than uniform across populations. Differences in adiposity distribution and metabolic characteristics across study cohorts may therefore contribute to the heterogeneity observed in the literature.

The specific biological pathways through which *FTO* influences breast cancer risk remain largely unclear, suggesting the involvement of multiple interacting factors^31^. One proposed mechanism is that *FTO* variation may influence breast cancer risk indirectly through its associations with body weight and metabolic function^35^. The gene’s association with obesity and T2D, together with reported associations with breast cancer, suggests involvement in metabolic pathways connecting adiposity and cancer risk^35^. Increased body weight is associated with expansion of adipose tissue, which functions as an endocrine organ essential for energy homeostasis. The expansion of adipose tissue alters the production of steroid hormones and adipokines, leading to metabolic disorders and subclinical inflammation^36^. These metabolic and inflammatory changes have also been implicated in carcinogenesis, tumor progression, and metastasis^36^. The rs9939609 SNP is located within intron 1 of the *FTO* gene and does not alter the structure of the *FTO* protein. Instead, it is thought to modulate *FTO* gene expression^37^. One proposed mechanism linking *FTO* variation to breast cancer risk involves altered regulation of the Iroquois homeobox 3 gene (*IRX3)*^38^, which plays a critical role in adipocyte differentiation and supports the established connection between FTO and obesity^38^. Moreover, *IRX3* has been implicated in breast cancer pathogenesis^39^. In addition to its regulatory effects on gene expression, *FTO* may contribute to cancer development through its enzymatic activity as an N6-methyladenosine (m6A) demethylase^40,41^. Given that m6A modifications influence RNA processing, stability, and translation^40,41^, alterations in *FTO*-mediated m6A demethylation may represent a mechanistic link between *FTO* and oncogenesis^37^.

However, although these cellular mechanisms raise the possibility of more direct effects of *FTO* variation on tumour biology, the ways in which these pathways interact with adiposity and metabolic factors to influence breast cancer risk in humans remain unclear. Our findings suggest that variation at the *FTO* locus may influence the association between substantial weight loss and breast cancer risk. Future studies should therefore evaluate breast cancer risk across *FTO* genotypes in the context of different weight-loss interventions, including emerging pharmacological treatments, and examine how these interventions influence metabolic, molecular, and cellular pathways relevant to breast cancer development.

A limitation of the SOS study is its non-randomized design. In the 1980s, due to concerns about high postoperative mortality rates, randomization was not considered acceptable. Also, some of the surgical treatments performed are less frequently used today, which may contribute to different treatment outcomes compared to current surgery techniques. In addition, several other limitations should be acknowledged. First, the study was not designed to evaluate the impact of the *FTO* gene on weight, insulin levels, and breast cancer. Second, the overall number of breast cancer events, as well as the number within genotype- and insulin-stratified subgroups was relatively modest which warrants cautious interpretation. Third, menopausal status was assessed only at baseline, and we could not distinguish between pre- and postmenopausal breast cancer at diagnosis, although obesity-related risk differs by menopausal status. Fourth, residual confounding cannot be excluded due to incomplete information on certain established breast cancer risk factors at baseline, including reproductive and family history. Fifth, tumour-specific characteristics (e.g., ER, PR, or HER2 status) were not available, precluding subtype-specific analyses. Sixth, information on mode of cancer detection was unavailable, and potential differences in screening patterns after substantial weight loss cannot be excluded, raising the possibility of detection bias. Finally, the cohort consists predominantly of individuals of European ancestry, which may limit generalizability to other populations. However, the study’s strengths include well-characterized participants, the possibility to compare bariatric surgery to a carefully matched control group receiving conventional obesity treatment, and an extensive follow-up period with access to comprehensive national registers, allowing for long-term cancer event tracking.

## Conclusions

In women with obesity, an association between bariatric surgery and reduced risk of breast cancer was observed among carriers of the *FTO* rs9939609 A‑allele. These findings suggest that genetic variation may modify the magnitude of cancer risk reduction achieved through substantial weight loss. Replication in independent cohorts and across different weight-loss modalities is warranted.

## Supplementary Information

Supplementary Information.

## Data Availability

The data is subject to legal restrictions according to national legislation. Confidentiality regarding personal information in studies is regulated in the Public Access to Information and Secrecy Act (SFS 2009:400). There is a possibility to apply to get access to public documents that an authority holds. In this case, the University of Gothenburg is the specific authority that holds the documents. A request to get access to public documents can be rejected or granted with reservations. If the authority refuses to disclose the documents the applicant is entitled to get a written decision that can be appealed to the administrative court of appeal.

## Abbreviations

BMI: Body mass index (kg/m^2^)
FTO: Fat mass and obesity-associated protein
PCR: Polymerase chain reaction
SOS: Swedish Obese Subjects (SOS)
SNP: Single nucleotide polymorphism
HR: Hazard ratio
T2D: Type 2 diabetes
CI: Confidence intervals
IRX3: Iroquois homeobox 3 gene
m6A: N6-methyladenosine demethylase
