# Supplementary material for: Association between FTO rs9939609 genotype and breast cancer risk after bariatric surgery in the Swedish Obese Subjects study
Source: Sci Rep. 2026 May 6;16:14429. doi: 10.1038/s41598-026-51884-2 (PMC13149690; doi:10.1038/s41598-026-51884-2)

Supplementary material

Association between *FTO* rs9939609 genotype and breast cancer risk after bariatric surgery in the Swedish Obese Subjects study

Elin Langegård, Felipe M. Kristensson, Johanna C. Andersson-Assarsson, Markku Peltonen, Per-Arne Svensson, Peter Jacobson, Sofie Ahlin, Kajsa Sjöholm, Lena M. S. Carlsson, Magdalena Taube

**Table of contents**	**Page**
sFigure 1. Cumulative incidence of breast cancer in the control group stratified by *FTO* rs9939609 genotype.	2
sFigure 2. Association between bariatric surgery and breast cancer incidence by *FTO* rs9939609 genotype, after additional adjustment for menopausal status and hormone replacement therapy (HRT) use at baseline.	3
sFigure 3. Association between *FTO* rs9939609 genotype and breast cancer incidence after bariatric surgery, evaluated using an additive genetic model.	4
sFigure 4. Association between bariatric surgery and breast cancer incidence by *FTO* rs9939609 genotype and baseline insulin level after exclusion of individuals with medically treated diabetes.	5
sFigure 5. Changes in insulin levels in women from the SOS study, stratified by treatment group and *FTO* rs9939609 genotype.	6

sFigure 1. Cumulative incidence of breast cancer in the control group stratified by FTO rs9939609 genotype.

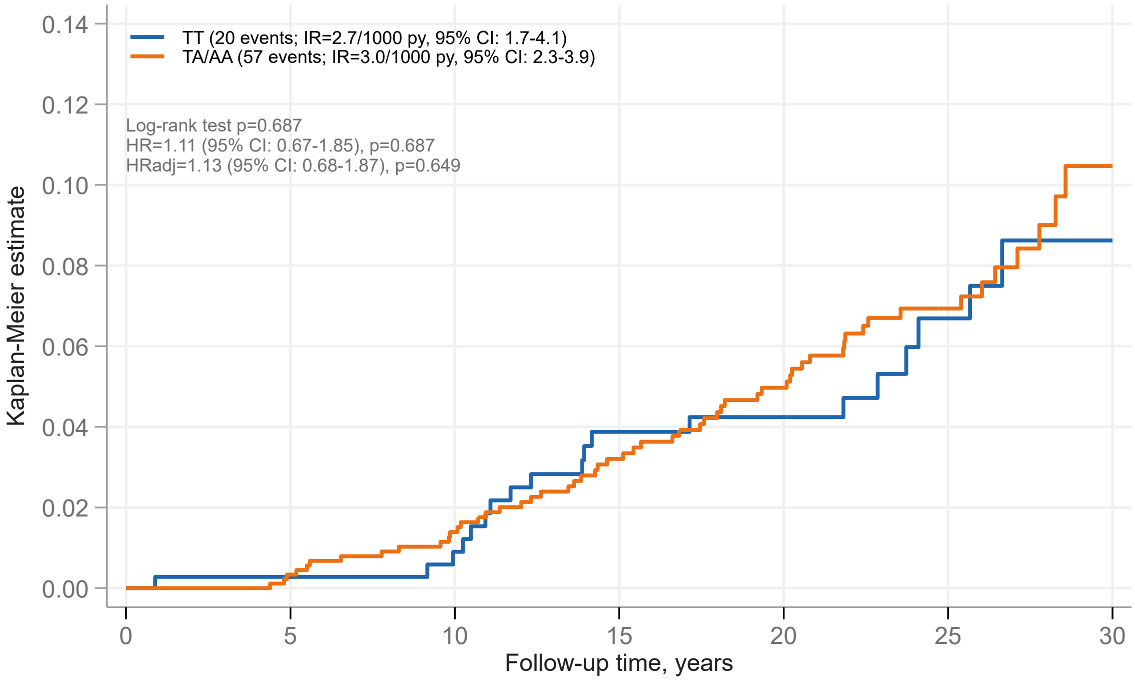

Number at risk							
TT	362	349	313	270	238	120	29
TA/AA	923	885	809	691	616	320	74

sFigure 2. Association between bariatric surgery and breast cancer incidence by *FTO* rs9939609 genotype, after additional adjustment for menopausal status and hormone replacement therapy (HRT) use at baseline.

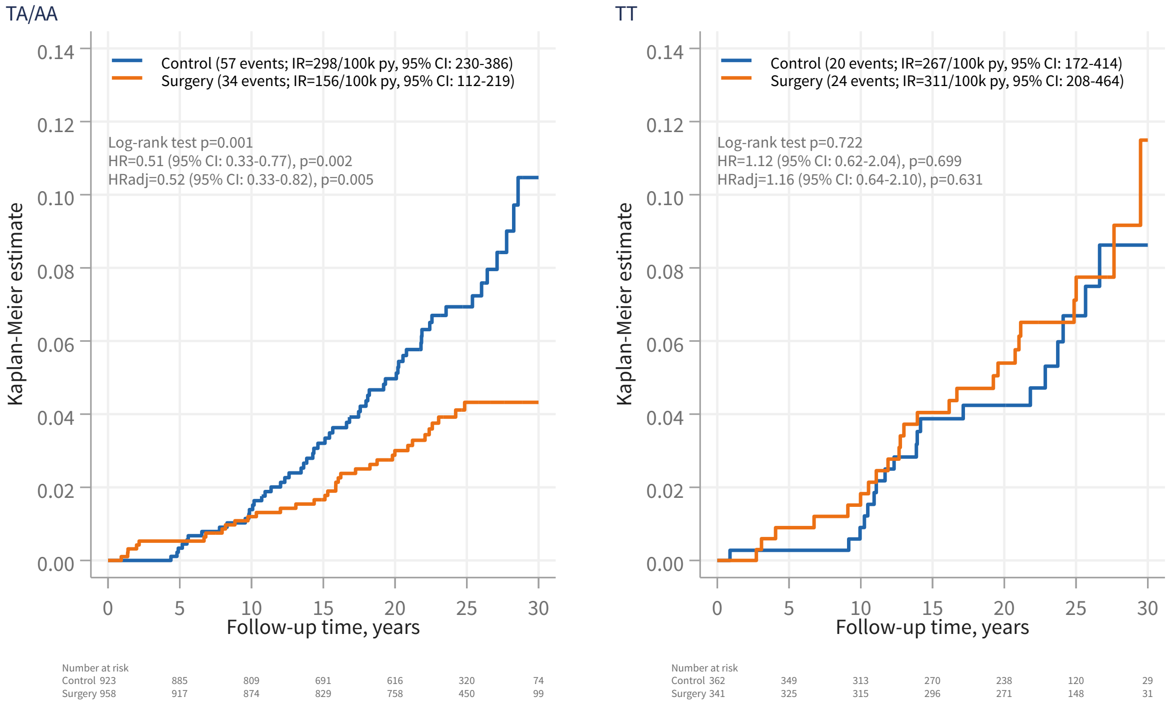

sFigure 3. Association between FTO rs9939609 genotype and breast cancer incidence after bariatric surgery, evaluated using an additive genetic model.

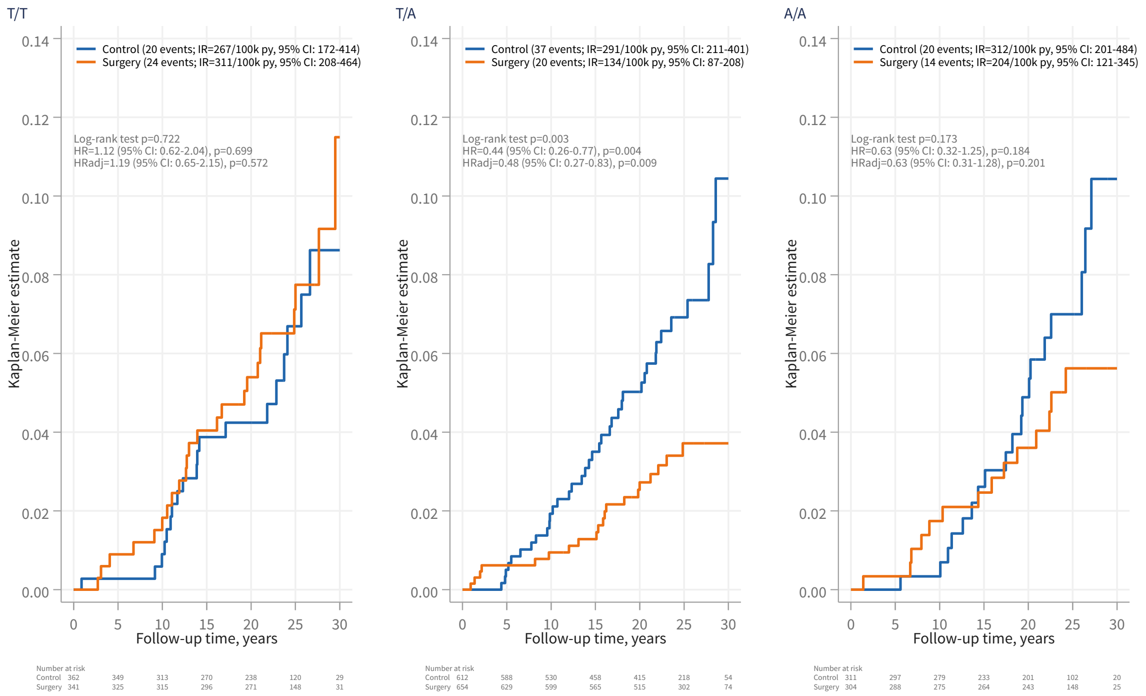

sFigure 4. Association between bariatric surgery and breast cancer incidence by FTO rs9939609 genotype and baseline insulin level after exclusion of individuals with medically treated diabetes.

Insulin low (<15.5 mU/L)

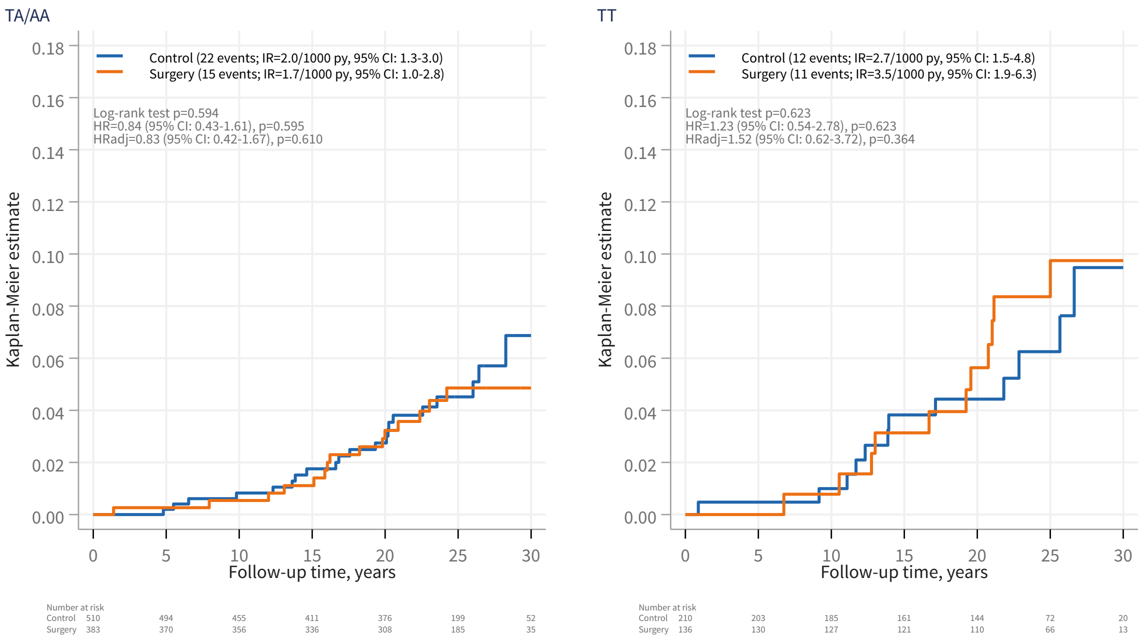

Insulin high (≥15.5 mU/L)

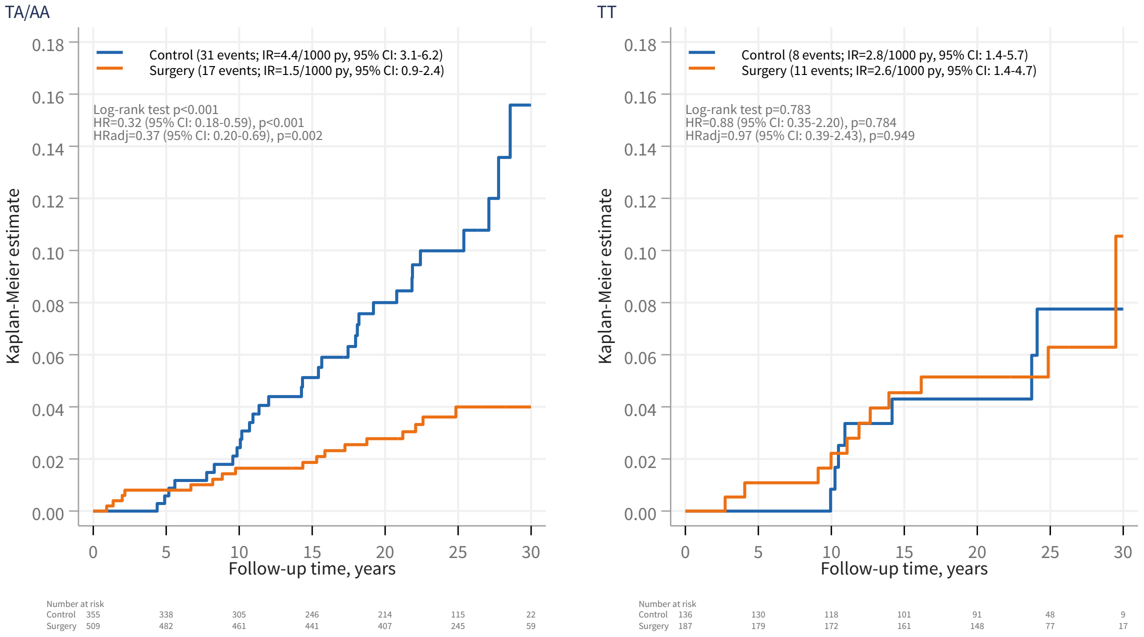

sFigure 5. Changes in insulin levels in women from the SOS study, stratified by treatment group and FTO rs9939609 genotype. Error bars represent 95 % CI.

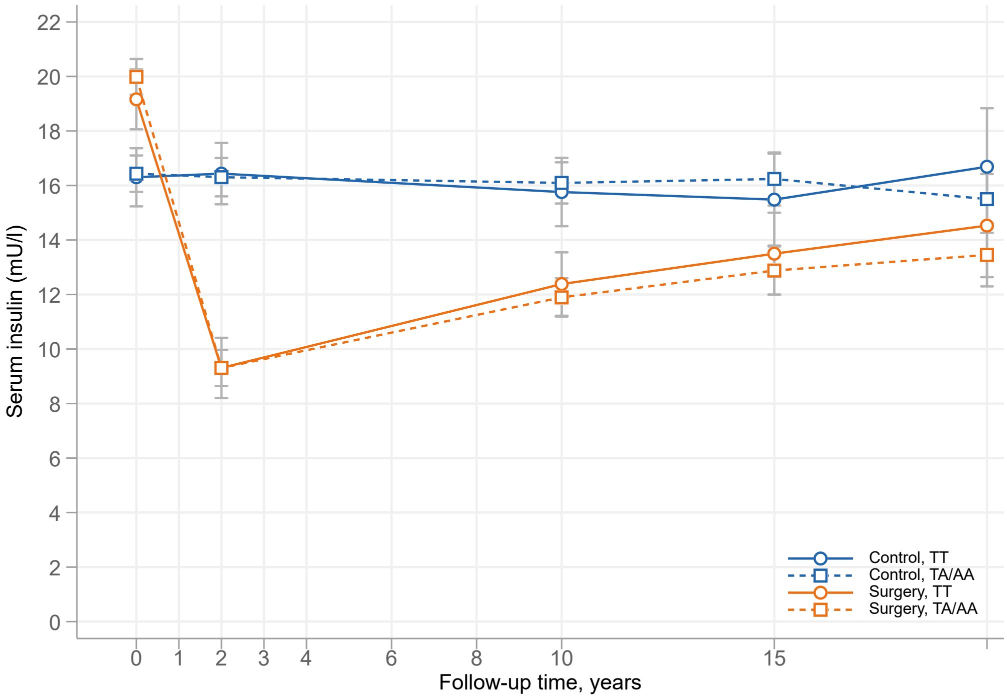

Supplement: Supplementary file 1 — Supplementary Information. [file 41598_2026_51884_MOESM1_ESM.pdf]
